# Psychosocial factors and attendance at a population-based mammography screening program in a cohort of Swedish women

**DOI:** 10.1186/1472-6874-14-33

**Published:** 2014-02-24

**Authors:** Magdalena Lagerlund, Jessica M Sontrop, Sophia Zackrisson

**Affiliations:** 1 Department of Clinical Sciences in Malmö, Diagnostic Radiology, Lund University, Skåne University Hospital Malmö, Inga Marie Nilssons gata 49, SE 20502 Malmö, Sweden; 2 Department of Epidemiology and Biostatistics, Western University, Kresge Building, Room K201, London, Ontario N6A 5C1, Canada

**Keywords:** Mammography, Breast cancer screening, Psychosocial factors, Social support, Sense of control, Stress

## Abstract

**Background:**

A better understanding of the factors that influence mammography screening attendance is needed to improve the effectiveness of these screening programs. The objective of the study was to examine whether psychosocial factors predicted attendance at a population-based invitational mammography screening program.

**Methods:**

Data on cohabitation, social network/support, sense of control, and stress were obtained from the Malmö Diet and Cancer Cohort Study and linked to the Malmö mammography register in Sweden. We analyzed 11,409 women (age 44 to 72) who were free of breast cancer at study entry (1992 to 1996). Mammography attendance was followed from cohort entry to December 31, 2009. Generalized Estimating Equations were used to account for repeated measures within subjects. Adjusted odds ratios (OR) and 95% confidence intervals (CI) are reported.

**Results:**

Among 69,746 screening opportunities there were 5,552 (8%) cases of non-attendance. Higher odds of non-attendance were found among women who lived alone (OR = 1.47 (1.33-1.63)) or with children only (OR = 1.52 (1.29-1.81)), had one childbirth (OR = 1.12 (1.01-1.24)) or three or more childbirths (OR = 1.34 (1.21-1.48)), had low social participation (OR= 1.21 (1.10-1.31)), low sense of control (OR = 1.12 (1.02-1.23)), and experienced greater stress (OR = 1.24 (1.13-1.36)).

**Conclusions:**

Public health campaigns designed to optimize mammography screening attendance may benefit from giving more consideration of how to engage with women who are less socially involved.

## Background

The public health impact of population-based screening programs depends to a large extent on optimizing participation rates. Attendance rates in Swedish counties have ranged between 66 and 91%, and tend to be lower in metropolitan regions [1,2]. A better understanding of the factors influencing attendance at outreach mammography screening programs is important for further improvement of the organization and effectiveness of mammography screening.

Following recommendations from the National Board of Health and Welfare, female residents of Malmö, Sweden (the setting of the current study) have been invited to a population-based mammography screening program since 1990, in intervals of 1.5 or 2 years depending on age group and breast density [3]. Between 1990 and 1993, coinciding with baseline data collection for the current study, 65% of the invited women attended screening [3], resulting in one of the lowest attendance rates in Sweden. The comparatively low screening attendance made it particularly interesting to study factors affecting screening attendance in this geographical area.

There is some evidence that psychosocial factors may affect mammography attendance. Being married or living with a partner, social support, and social participation are all associated with greater mammography attendance [3-10]; and social isolation and poor sense of control are associated with lower attendance [9,11]. It is possible that psychosocial variables influence attendance through various mechanisms including social norms, self-efficacy, and perceived sense of responsibility towards family and/or society to take care of oneself. Whereas social networks may provide practical, financial, emotional and social support, which may facilitate preventive health actions, experiencing stress and lacking a sense of control could impede women’s motivation to take health actions. Furthermore, civic and social participation can empower individuals and groups of individuals to take responsibility and control over their own lives [12,13].

While marital status and cohabitation have previously been examined in relation to mammography screening attendance in Sweden [3,5,6], other psychosocial aspects (e.g. perceived social support, social engagement, stress and sense of control) have not. These latter factors have been examined in relation to mammography to some extent in other countries [9,11,14-16] but more commonly in relation to cancer incidence and survival [17-23].

We undertook the present study to examine the relationships between psychosocial factors and mammography screening attendance among Swedish women between 1992 and 2009. In particular, we examined family factors (cohabitation, parity, and siblings), loneliness, social participation, social anchorage, instrumental and emotional support, sense of control, and stress.

## Methods

### Design, setting, and population

In this community-based cohort study, we linked data from the *Malmö Diet and Cancer Study* (MDCS) to the *Malmö Mammographic Screening Register*. Data sources and sample selection are described below. This study was approved by the ethics committee at Lund University.

### Data sources

The *Malmö Diet and Cancer Study* (MDCS) is a prospective cohort study that is investigating associations between diet and cancer. Recruitment began in 1991 and was predominantly done by postal invitation at random from the source population of Malmö residents born between 1926 and 1945. An additional 18.1% of the respondents joined the study spontaneously as a result of the passive recruitment campaign, and recruitment was extended to some older and younger age groups in 1995. At the end of recruitment in the autumn of 1996, a total of 17,035 women had joined the study [24]. At baseline, all participants completed a health questionnaire, which was reviewed for missing answers by a research assistant during the second study visit, a few weeks later.

The *Malmö Mammographic Screening Program* is an outreach mammography screening program that was established in Malmö, Sweden in 1990 [3]. Malmö is located in southern Sweden, population 307,758 in 2012 [25]. Invitations to attend mammography screening are mailed to all eligible female residents in intervals of 1.5-2 years. Reminders are not used. The mammography register is continuously updated to ensure inclusion of all eligible women (all Swedish residents are assigned a unique 10-digit personal identification number that can be linked to a central health and census register). The screening register contains basic information on number of invitations, dates, attendance, and recall for each woman. The age groups invited have varied somewhat over the years due to changes in recommendations. Between 1990 and 1998, only women aged 50–69 were invited; however, in 1999 the upper age limit was extended to 74 years, and since 2009 the lower age limit decreased to 40 years. Two-view mammography and double reading of the mammograms is practiced.

### Sample

**Figure 1:**
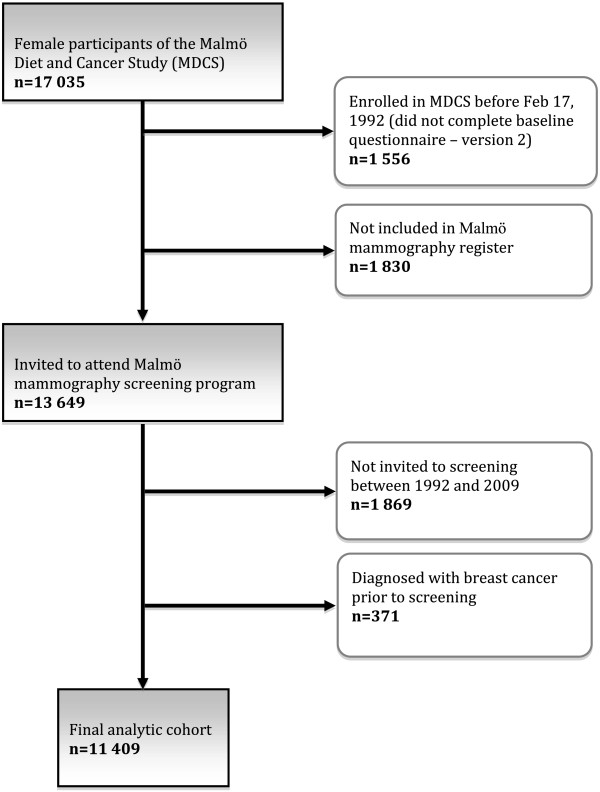
**Selection of analytic cohort.** With kind permission from Springer Science+Business Media: Cancer Causes Control, Are reproductive and hormonal risk factors for breast cancer associated with attendance at mammography screening? 24, 2013, 1687-94, Lagerlund M, Sontrop J, Zackrisson S, figure 1.

For the purpose of this study, we selected women who completed the MDCS baseline questionnaire between Feb 17, 1992 and Sept 25, 1996 (second and third questionnaire versions), and who had been invited to the mammographic screening program in Malmö between baseline and end of follow-up (date of death, date of emigration from Sweden, date of breast cancer diagnosis, or Dec 31, 2009, whichever came first). We excluded women who had been diagnosed with breast cancer before the baseline interview. The sample flow chart in Figure 1 describes the different steps of exclusion, resulting in a final sample of 11,409 individuals.

### Measures and definitions

#### Mammography screening attendance

The outcome variable of interest in this study is mammography screening non-attendance and we used the individual mammography invitation as the unit of analysis. Among the 11,409 subjects included in the study sample there was a total of 69,746 screening opportunities (invitations) during follow-up. Of these, 64,194 (92%) resulted in attendance and 5,552 (8%) resulted in non-attendance.

#### Psychosocial factors

Information on psychosocial factors was obtained from the baseline MDCS health questionnaire. We examined the following familial factors: *cohabitation* (living alone versus living with partner only, with partner and children, with children only, or with parents or other), *siblings* (yes or no), and number of *childbirths* (none, one, two or three or more). Information on adopted children, stepchildren, or other children was not available.

We examined seven other psychosocial factors. Participants’ degree of *social participation* was based on responses to 13 items describing participation in formal and informal social groups and activities (participation in a study circle/course at work or outside of work, or meetings held by unions or other organizations, writing a letter to editor of a newspaper/journal, attending theatre/cinema, art exhibitions, church, night club/entertainment, private parties, sports events, demonstrations or gathering with relatives). Participants with affirmative responses to three or fewer of these activities were considered to have low social participation [26]. Participants’ degree of *social anchorage* was based on responses to five items describing belonging, anchorage, and feeling of membership/solidarity in formal and informal groups, and rootedness in community. If three or more of the five items denoted low social anchorage, overall social anchorage was categorized as low [26]. Participants were considered to have good *instrumental support* if they indicated high certainty about having access to practical help and support from other people (e.g., during illness) [26]. Participants’ degree of *emotional support* was based on responses to three questions describing availability of people “to talk to about most things”, “get support from in difficult situations in life”, and “to be yourself with”. Overall emotional support was categorized as low if participants indicated having low support for ≥2 items. Participants were also asked how often they had *feelings of loneliness* (often/sometimes versus seldom/never). Participants’ sense of *control/mastery* was based on responses to four questions about control over important things in life, personal problems, and things not turning out the way one had wished and was categorized as low if lack of control was experienced fairly to very often for ≥3 items. Finally, participants were asked if they had experienced recent *stress or mental pressure outside of work* (yes/no).

### Statistical analysis

Descriptive characteristics are reported as mean (standard deviation (SD)) or count (percent). Reference categories for predictor variables were chosen based on the greatest likelihood of non-attendance (from unadjusted cross-tabulations with the outcome). We estimated odds ratios (OR) and 95% confidence intervals (CI) for mammography attendance from binary Generalized Estimating Equation (GEE) models where adjustments are made for correlation of repeated measures within subjects (with an autoregressive correlation structure). There were only repeated measures for the outcome variable and not for the predictor variables. We first conducted bivariate analyses to examine the effect of each psychosocial factor on the odds of non-attendance, and then adjusted the estimates by age at baseline. Multivariate analyses were conducted in two steps. In the first step (Model 1) we adjusted each psychosocial variable for the set of sociodemographic and screening variables (age, education, employment status, and country of birth, number invitations received after baseline, and having been invited to screening before baseline). In a second step all psychosocial variables were entered simultaneously along with the sociodemographic and screening variables (Model 2).

## Results

Of 17,035 women who completed the MCDS baseline questionnaire between 1992 and 1996, 13,649 received at least one invitation to attend the Malmö mammography screening clinic before December 31, 2009, and 11,409 met our inclusion criteria (Figure 1). Among all the screening invitations that were sent to the study sample throughout the follow-up period 92.0% resulted in attendance, with variations between 87.6% and 94.5% in different calendar years (data not shown). The number of screening opportunities after baseline ranged between one and twelve, and there was a positive association between the number of invitations received and screening attendance. The more invitations received the higher the odds of attending (data not shown).

Sample characteristics are presented in Table 1. At baseline, women were an average of 54.9 (6.7) years (range: 44 to 72). Mean age at first screening opportunity was 56.7 (6.4) years (range: 45 to 75). Approximately two-thirds of women had not completed high school, more than two-thirds were employed, and 88.3% were born in Sweden. In terms of occupational status (present or most recent position), 55.6% were non-manual workers and 36.6% were manual workers. Table 2 shows the distribution of the psychosocial factors in the study sample.

**Table 1:** Sociodemographic and screening characteristics of study sample (n = 11,409), Malmö, Sweden (1992–2009)

**Sociodemographic variables**	**N (%)**
Mean age at baseline (SD)	54.9 (6.7)
Mean age at 1st subsequent screening invitation (SD)	56.7 (6.4)
Age group at baseline (years)	
44–49	3,442 (30.2)
50–54	2,470 (21.6)
55–59	2,086 (18.3)
60–64	2,155 (18.9)
65–72	1,256 (11.0)
Education level	
High school or higher	3,717 (32.7)
Less than high school	7,665 (67.3)
Missing	27
Occupation (present or latest job)	
Self-employed/employer/farmer	885 (7.8)
Higher non-manual	741 (6.6)
Middle non-manual	2,049 (18.1)
Lower non-manual	3,489 (30.9)
Skilled manual	803 (7.1)
Unskilled manual	3,341 (29.5)
Missing	101
Employment status	
Employed	7,695 (67.6)
Not employed	3,681 (32.4)
Missing	33
Country of birth	
Sweden	10,066 (88.3)
Other	1,336 (11.7)
Missing	7
Invited to screening program before baseline	
Yes	8,459 (74.1)
No	2,950 (25.9)
Number of screening invitations after baseline	
1	793 (7.0)
2	602 (5.3)
3	568 (5.0)
4	808 (7.1)
5	1,271 (11.1)
6	1,626 (14.3)
7	1,896 (16.6)
8	1,921 (16.8)
9	1,247 (10.9)
10	609 (5.3)
11	67 (0.6)
12	1 (0.0)

**Table 2:** Distribution of psychosocial factors in study sample (n = 11,409), Malmö, Sweden (1992–2009)

**Psychosocial factors**	**N (%)**
Living alone or with others	
Alone	2,851 (25.0%)
With partner only	5,549 (48.7%)
With partner and children	2,322 (20.4%)
With children only	576 (5.1%)
With parents or other	104 (0.9%)
Number of children	
None	1,541 (13.5%)
One	2,365 (20.8%)
Two	4,848 (42.6%)
Three or more	2,632 (23.1%)
Having siblings	
Yes	9,929 (87.0%)
No	1,480 (13.0%)
Feeling lonely	
Often/sometimes	4,619 (40.6%)
Seldom/never	6,763 (59.4%)
Missing	27
Social participation (13 items)	
Low	3,128 (27.5%)
High	8,252 (72.5%)
Missing	29
Social anchorage (5 items)	
Low	1,044 (9.2%)
High	10,358 (90.8%)
Missing	7
Instrumental support (1 item)	
Low (low/medium)	2,831 (24.9%)
High	8,557 (75.1%)
Missing	24
Emotional support (3 items)	
Low	2,298 (20.2%)
High	9,097 (79.8%)
Missing	14
Control/Mastery (4 items)	
Low	3,294 (29.0%)
High	8,056 (71.0%)
Missing	59
Stress (non-work related)	
Yes	3,830 (33.7%)
No	7,548 (66.3%)
Missing	31

Associations between psychosocial factors and mammography non-attendance are presented in Table 3. Most effects weakened with additional adjustments. The results of the multivariate analysis (Model 2) showed a statistically significant association between non-attendance and living alone (OR = 1.47, 95% CI: 1.33-1.63) or with children only (OR=1.52, 95% CI: 1.29-1.81). In the small group of women who lived with parents or other non-attendance was more common, but this association was not quite statistically significant (OR = 1.44, 95% CI: 0.99-2.10). Number of childbirths was associated with mammography attendance, but the relationship was not linear – while high mammography attendance was most common among women with two children, having one child or three or more children was significantly associated with non-attendance (OR = 1.12, 95% CI: 1.01-1.24 and OR = 1.34, 95%: 1.21-1.48, respectively). In general, social network and support factors were positively related to mammography attendance, but after controlling for other factors only low social participation predicted non-attendance (OR = 1.21, 95% CI: 1.10-1.31). The odds of non-attendance were also greater among those who had a low sense of control/mastery (OR = 1.12, 95% CI: 1.02-1.23), and those who experienced greater non-work related stress (OR = 1.24, 95% CI: 1.13-1.36).

**Table 3:** Psychosocial factors (social network/support, control and stress) in relation to non-attendance at mammography screening, Malmö, Sweden (1992–2009)

	**Odds ratios for non-attendance (95% CI)**			
**Psychosocial factors**	**Unadjusted**	**Age adjusted**	**Multivariate model 1**^**§**^	**Multivariate model 2**^**†**^
Living alone or with others				
Alone	1.61 (1.46–1.76)***	1.59 (1.45–1.75)***	1.54 (1.40–1.69)***	1.47 (1.33–1.63)***
With partner only	Ref	Ref	Ref	Ref
With partner and children	1.23 (1.11–1.36)***	1.07 (0.96–1.20)	1.00 (0.89–1.13)	0.94 (0.84–1.06)
With children only	2.26 (1.93–2.64)***	1.96 (1.66–2.31)***	1.75 (1.48–2.06)***	1.52 (1.29–1.81)***
With parents or other	1.63 (1.09–2.42)*	1.56 (1.05–2.31)*	1.47 (1.00–2.15)	1.44 (0.99–2.10)
Number of children				
None	1.08 (0.95–1.22)	1.08 (0.96–1.23)	1.05 (0.93–1.19)	0.95 (0.83–1.08)
One	1.22 (1.10–1.36)***	1.24 (1.11–1.37)***	1.19 (1.08–1.32)***	1.12 (1.01–1.24)*
Two	Ref	Ref	Ref	Ref
Three or more	1.39 (1.26–1.53)***	1.42 (1.29–1.57)***	1.37 (1.24–1.51)***	1.34 (1.21–1.48)***
Having siblings				
Yes	Ref	Ref	Ref	Ref
No	1.08 (0.96–1.22)	1.08 (0.96–1.22)	1.07 (0.95–1.21)	1.05 (0.93–1.18)
Feeling lonely				
Often/sometimes	1.36 (1.26–1.47)***	1.36 (1.26–1.47)***	1.30 (1.20–1.40)***	1.02 (0.93–1.12)
Seldom/never	Ref	Ref	Ref	Ref
Social participation (13 items)				
Low	1.27 (1.16–1.38)***	1.34 (1.23–1.46)***	1.28 (1.17–1.41)***	1.21 (1.10–1.33)***
High	Ref	Ref	Ref	Ref
Social anchorage (5 items)				
Low	1.30 (1.15–1.47)***	1.28 (1.13–1.45)***	1.19 (1.06–1.34)**	1.01 (0.89–1.14)
High	Ref	Ref	Ref	Ref
Instrumental support (1 item)				
Low (low/medium)	1.17 (1.07–1.28)***	1.18 (1.08–1.29)***	1.17 (1.07–1.28)***	1.02 (0.92–1.12)
High	Ref	Ref	Ref	Ref
Emotional support (3 items)				
Low	1.28 (1.17–1.40)***	1.30 (1.18–1.42)***	1.26 (1.15–1.39)***	1.09 (0.98–1.21)
High	Ref	Ref	Ref	Ref
Control/Mastery (4 items)				
Low	1.44 (1.33–1.56)***	1.42 (1.31–1.54)***	1.33 (1.22–1.44)***	1.12 (1.02–1.23)*
High	Ref	Ref	Ref	Ref
Stress (non-work related)				
Yes	1.53 (1.41–1.66)***	1.49 (1.38–1.61)***	1.40 (1.29–1.52)***	1.24 (1.13–1.36)***
No	Ref	Ref	Ref	Ref

*p < 0.05, **p < 0.01, ***p < 0.001.

^§^Adjusted for age, education, employment status, country of birth, number of invitations received, and having been invited to screening before baseline.

^†^Adjusted for all other psychosocial factors along with age, education, employment status, country of birth, number of invitations received, and having been invited to screening before baseline.

## Discussion

In this cohort of Swedish women who received regular invitations to attend mammography screening, non-attendance was more common among women who were living alone, among single mothers, among women with one child or three or more children, and among those who had low social participation, low control and greater stress.

In line with a previous Swedish study [5] the present study showed that women not living with a partner were less likely to attend mammography screening. However, a Canadian study did not find a significant association between living with a partner and having had a mammogram in the previous year [27]. Having a partner may increase the likelihood of being proactive about one’s health, perhaps due to feeling accountable towards a significant other.

Although nulliparity is a known risk factor for breast cancer, we observed a non-linear relationship between mammography attendance and parity. Non-attendance was more common among women who had given birth to one child or three or more children compared with women who had two children, and nulliparous women did not differ significantly from those with two children. The literature shows a similar mixed picture [5,28], with many studies finding no effect of nulliparity on screening [29-33], a positive relationship between parity and screening [6,34,35], or an inverse or U-shaped association between parity and screening [5,28,36-38]. While having dependent children may increase motivation to care for one’s health, having multiple children may impose time constraints. High-parity women were more likely to have children living at home with them (33.4%), compared with 21.8% among those with only one child (data not shown). Adjusting for age, cohabitation, stress and other covariates did not appreciably affect the association between high parity and lower mammography attendance.

Social participation appears to capture a different aspect of social life than that represented by familial factors such as cohabitation and parity. In contrast to familial support, social participation indicates how socially active and engaged women are outside the home. As shown in multivariate model 2, the effect of social participation was not appreciably affected by the presence of other social network variables or familial factors. A social network may encourage greater screening attendance or other preventive health care, perhaps through perceived sense of responsibility towards one’s social group to take care of oneself or through social pressures to follow prevailing social norms around screening. As demonstrated in other studies, mammography screening is positively associated with general social support [9,15], having a close friend [4], social support from significant others (e.g. spouse, friends, health professional) [7,38], being a member of a volunteer group [8,9], and inversely associated with social isolation [11]. However, in a US study, no association was evident between ever having had a mammographic screening test and a social network index variable [39]. In the present study, social participation was the only other social network factor that remained statistically significant after adjusting for cohabitation and number of children.

In the final multivariate model sense of control was still associated with screening attendance. The effect was small but still statistically significant. Our finding corroborates that of a Canadian study where women with moderate or low sense of control were found to be more likely to report never having had a mammogram compared to women with high sense of control [9]. Believing that circumstances in life are caused by factors outside of one’s control may lead to a less preventive approach to health.

Stress or distress has mostly been studied in association with the mammography screening examination itself or in relation to waiting for or dealing with the results of the screening test. In contrast, the present study examined stress as a reflection of the women’s general life situation and its impact on screening attendance. One explanation for a negative effect of stress on screening attendance may be that women who experience higher levels of stress have less time or energy for attending screening tests. However, this result contradicts findings from other studies. A Finnish study found that stress was unrelated to mammography attendance [14], and an American study found that women who experienced high stress in the past year were more likely to have had a mammogram compared with women who experienced low stress [16]. Differences across studies may result from differing definitions of stress and differing cultural experiences of stress.

Overall, our findings are in agreement with several other studies which show that mammography screening is lower among women who are more socially isolated, who are not married or cohabitating, and who participate in few social activities. If this group is under-screened, they may have a greater risk of undetected breast cancer and possibly an increased risk for death from breast cancer. Most research on social support and cancer mortality has been conducted in the US. A prospective study found social isolation to be associated with worse breast cancer survival, but did not find an effect of involvement in community activities [19]. Lower breast cancer survival has been found among women who were single, separated, divorced or widowed than among married women [40]. Other studies have not found an effect of social isolation on breast cancer mortality or incidence. [17,20,41,42].

### Strengths and limitations

The sample constituted of a selection of individuals with high screening attendance. This study cohort had an overall mammography attendance rate of 92%, which is considerably higher than the 65% observed in the general population of this geographical area at the time of the baseline data collection [3]. This is likely due to the fact that the MDCS cohort has a higher proportion of Swedish born women, is selected towards better health, and possibly higher socio-economic status and healthier lifestyle than the general population [43]. Although it is possible that the distribution of psychosocial factors may differ among non-responders (the proportions could be either higher or lower), and psychosocial factors may affect mammography attendance differently in the general population, our findings are consistent with a large body of literature showing an inverse association between social support, social participation and mammography attendance. The psychosocial measures included in our analysis were limited to variables available in the MDCS. Questions on social support and participation were not tailored towards preventive health measures or health concerns in particular; however, many aspects of social network and support were captured, including social participation and anchorage, and both emotional and instrumental support. Because of the prospective nature of this study and long follow-up period (median follow-up: 15 years; range: 4–18), it is possible that women’s psychosocial situation may have changed between baseline and screening. However, this type of exposure misclassification would likely attenuate rather than inflate associations with the outcome. The strengths of this study include its large size and prospective follow-up of screening attendance. While other studies largely relied on self-reported mammography attendance, our measure came from register data, which would reduce the risk of outcome misclassification. Further, information on psychosocial factors was obtained independently of mammography screening. Finally, since we had register information on breast cancer diagnoses from the cancer register we excluded women who had had breast cancer before baseline and were thus able to examine screening attendance prior to breast cancer diagnosis.

## Conclusions

In this Swedish cohort of over 11,000 women who received regular invitations to attend mammography screening, non-attendance was associated with living alone, single motherhood, having given birth to one child or three or more children, low social participation, low control/mastery, and greater stress. The results of our analysis provide some support for the argument that public health campaigns designed to optimize mammography screening attendance may benefit from giving more consideration of how to engage with women who are less socially involved. Future research should evaluate whether socially isolated women are at greater risk for undetected breast cancer.

## Competing interests

The authors declare that they have no competing interests.

## Authors’ contributions

ML was involved in research proposal development, planning and analysis of data and preparation of the manuscript. JMS made a substantial contribution to the interpretation of results and preparation of the manuscript. SZ was the founder of the research idea, developed the research proposal, and was involved in data analysis and revision of the manuscript. All authors read and approved the final manuscript.

## Pre-publication history

The pre-publication history for this paper can be accessed here:

http://www.biomedcentral.com/1472-6874/14/33/prepub

## References

[B1] Olsson S Andersson I Karlberg I Bjurstam N Frodis E Hakansson S Implementation of service screening with mammography in Sweden: from pilot study to nationwide programme J Med Screen 2000 7 1 14 18 10.1136/jms.7.1.14 10807141

[B2] Swedish Association of Local Authorities and Regions Insatser för tidig upptäckt. Ännu bättre cancervård - delrapport 4 2013 Stockholm, Sweden: Swedish Association of Local Authorities and Regions http://www.skl.se/vi_arbetar_med/halsaochvard/cancervard/slutrapportering-2013/delrapporter-2013?MA_START_FOLDER=74c0f39d-a8ba-487d-850f-5cd7b9222cd2.

[B3] Zackrisson S Andersson I Manjer J Janzon L Non-attendance in breast cancer screening is associated with unfavourable socio-economic circumstances and advanced carcinoma Int J Cancer 2004 108 5 754 760 10.1002/ijc.11622 14696103

[B4] Calnan M The health belief model and participation in programmes for the early detection of breast cancer: a comparative analysis Soc Sci Med 1984 19 8 823 830 10.1016/0277-9536(84)90399-X 6505748

[B5] Lagerlund M Maxwell AE Bastani R Thurfjell E Ekbom A Lambe M Sociodemographic predictors of non-attendance at invitational mammography screening–a population-based register study (Sweden) Cancer Causes Control 2002 13 1 73 82 10.1023/A:1013978421073 11899121

[B6] Lagerlund M Sparen P Thurfjell E Ekbom A Lambe M Predictors of non-attendance in a population-based mammography screening programme; socio-demographic factors and aspects of health behaviour Eur J Cancer Prev 2000 9 1 25 33 10.1097/00008469-200002000-00004 10777007

[B7] Lechner L de Vries H Offermans N Participation in a breast cancer screening program: influence of past behavior and determinants on future screening participation Prev Med 1997 26 4 473 482 10.1006/pmed.1997.0161 9245669

[B8] Maxwell CJ Bancej CM Snider J Predictors of mammography use among Canadian women aged 50–69: findings from the 1996/97 National Population Health Survey CMAJ 2001 164 3 329 334 11232132 PMC80725

[B9] Maxwell CJ Kozak JF Desjardins-Denault SD Parboosingh J Factors important in promoting mammography screening among Canadian women Can J Public Health 1997 88 5 346 350 9401172 10.1007/BF03403903 PMC6990268

[B10] Cabeza E Esteva M Pujol A Thomas V Sanchez-Contador C Social disparities in breast and cervical cancer preventive practices Eur J Cancer Prev 2007 16 4 372 379 10.1097/01.cej.0000236243.55866.b0 17554211

[B11] Hunt SM Alexander F Roberts MM Attenders and non-attenders at a breast screening clinic: a comparative study Public Health 1988 102 1 3 10 10.1016/S0033-3506(88)80004-0 3344310

[B12] Baum FE Bush RA Modra CC Murray CJ Cox EM Alexander KM Potter RC Epidemiology of participation: an Australian community study J Epidemiol Community Health 2000 54 6 414 423 10.1136/jech.54.6.414 10818116 PMC1731693

[B13] Israel BA Checkoway B Schulz A Zimmerman M Health education and community empowerment: conceptualizing and measuring perceptions of individual, organizational, and community control Health Educ Q 1994 21 2 149 170 10.1177/109019819402100203 8021145

[B14] Aro AR de Koning HJ Absetz P Schreck M Psychosocial predictors of first attendance for organised mammography screening J Med Screen 1999 6 82 88 10.1136/jms.6.2.82 10444726

[B15] Farmer D Reddick B D’Agostino R Jackson SA Psychosocial correlates of mammography screening in older African American women Oncol Nurs Forum 2007 34 1 117 123 10.1188/07.ONF.117-123 17562638

[B16] Rakowski W Rimer BK Bryant SA Integrating behavior and intention regarding mammography by respondents in the 1990 national health interview survey of health promotion and disease prevention Public Health Rep 1993 108 5 605 624 8210259 PMC1403437

[B17] Butow PN Hiller JE Price MA Thackway SV Kricker A Tennant CC Epidemiological evidence for a relationship between life events, coping style, and personality factors in the development of breast cancer J Psychosom Res 2000 49 3 169 181 10.1016/S0022-3999(00)00156-2 11110988

[B18] Chida Y Hamer M Wardle J Steptoe A Do stress-related pscychosocial factors contribute to cancer incidence and survival? Nat Clin Pract Oncol 2008 5 8 466 475 10.1038/ncponc1134 18493231

[B19] Kroenke CH Kubzansky LD Schernhammer ES Holmes MD Kawachi I Social networks, social support, and survival after breast cancer diagnosis J Clin Oncol 2006 24 7 1105 1111 10.1200/JCO.2005.04.2846 16505430

[B20] Reynolds P Kaplan GA Social connections and risk for cancer: prospective evidence from the Alameda county study Behav Med 1990 16 3 101 110 10.1080/08964289.1990.9934597 2224168

[B21] Santos MC Horta BL Amaral JJ Fernandes PF Galvao CM Fernandes AF Association between stress and breast cancer in women: a meta-analysis Cadernos de saude publica / Ministerio da Saude, Fundacao Oswaldo Cruz, Escola Nacional de Saude Publica 2009 25 Suppl 3 S453 S463 10.1590/s0102-311x2009001500010 20027392

[B22] Stringhini S Berkman L Dugravot A Ferrie JE Marmot M Kivimaki M Singh-Manoux A Socioeconomic status, structural and functional measures of social support, and mortality: the British Whitehall II cohort study, 1985–2009 Am J Epidemiol 2012 175 12 1275 1283 10.1093/aje/kwr461 22534202 PMC3372313

[B23] Surtees PG Wainwright NW Luben R Khaw KT Day NE Mastery, sense of coherence, and mortality: evidence of independent associations from the EPIC-Norfolk prospective cohort study Health Psychol 2006 25 1 102 110 16448303 10.1037/0278-6133.25.1.102

[B24] Manjer J Elmstahl S Janzon L Berglund G Invitation to a population-based cohort study: differences between subjects recruited using various strategies Scand J Public Health 2002 30 2 103 112 10.1177/14034948020300020401 12028859

[B25] Statistics Sweden Population by municipality, marital status and sex. Year 1968–2012 Population by region, marital status, age and sex. Year 1968 - 2012 http://www.scb.se/en_/Finding-statistics/Statistical-Database/Select-variables/?px_tableid=ssd_extern%3aBefolkningNy&rxid=31393f4d-66e1-4498-ae9a-7d02ad6c5261.

[B26] Lindstrom M Hanson BS Ostergren PO Berglund G Socioeconomic differences in smoking cessation: the role of social participation Scand J Public Health 2000 28 3 200 208 10.1080/140349400444904 11045752

[B27] Qi V Phillips SP Hopman WM Determinants of a healthy lifestyle and use of preventive screening in Canada BMC Public Health 2006 6 275 10.1186/1471-2458-6-275 17090313 PMC1636639

[B28] Flamant C Gauthier E Clavel-Chapelon F Determinants of non-compliance to recommendations on breast cancer screening among women participating in the French E3N cohort study Eur J Cancer Prev 2006 15 1 27 33 10.1097/01.cej.0000180666.11958.60 16374226 PMC2756596

[B29] Beaulieu MD Beland F Roy D Falardeau M Hebert G Factors determining compliance with screening mammography CMAJ 1996 154 9 1335 1343 8616736 PMC1487722

[B30] Donato F Bollani A Spiazzi R Soldo M Pasquale L Monarca S Lucini L Nardi G Factors associated with non-participation of women in a breast cancer screening programme in a town in northern Italy J Epidemiol Community Health 1991 45 1 59 64 10.1136/jech.45.1.59 2045747 PMC1060703

[B31] Paskett ED Tatum CM Mack DW Hoen H Case LD Velez R Validation of self-reported breast and cervical cancer screening tests among low-income minority women Cancer Epidemiol Biomarkers Prev 1996 5 9 721 726 8877064

[B32] Sutton S Bickler G Sancho-Aldridge J Saidi G Prospective study of predictors of attendance for breast screening in inner London J Epidemiol Community Health 1994 48 1 65 73 10.1136/jech.48.1.65 8138773 PMC1059897

[B33] Taplin S Anderman C Grothaus L Breast cancer risk and participation in mammographic screening Am J Public Health 1989 79 11 1494 1498 10.2105/AJPH.79.11.1494 2817159 PMC1349799

[B34] Bare ML Montes J Florensa R Sentis M Donoso L Factors related to non-participation in a population-based breast cancer screening programme Eur J Cancer Prev 2003 12 6 487 494 10.1097/00008469-200312000-00007 14639126

[B35] Staniscia T Manzoli LM Di Giovanni P Dragani V Testa P Cavaliere D Rauti I Schioppa F Romano F Factors related to the uptake of breast cancer screening (mammography and breast ultrasound): a retrospective survey on a sample of resident women, 50–70 years aged, from Abruzzo region Ann Ig 2003 15 6 1063 1075 15049564

[B36] Burton MV Warren R Price D Earl H Psychological predictors of attendance at annual breast screening examinations Br J Cancer 1998 77 11 2014 2019 10.1038/bjc.1998.335 9667685 PMC2150324

[B37] Caleffi M Ribeiro RA Bedin AJ Jr Viegas-Butzke JM Baldisserotto FD Skonieski GP Giacomazzi J Camey SA Ashton-Prolla P Adherence to a breast cancer screening program and its predictors in underserved women in southern Brazil Cancer Epidemiol Biomarkers Prev 2010 19 10 2673 2679 10.1158/1055-9965.EPI-10-0338 20716620

[B38] Seow A Straughan PT Ng EH Emmanuel SC Tan CH Lee HP Factors determining acceptability of mammography in an Asian population: a study among women in Singapore Cancer Causes Control 1997 8 5 771 779 10.1023/A:1018439623384 9328200

[B39] Bostick RM Sprafka JM Virnig BA Potter JD Predictors of cancer prevention attitudes and participation in cancer screening examinations Prev Med 1994 23 6 816 826 10.1006/pmed.1994.1139 7855115

[B40] Lai H Lai S Krongrad A Trapido E Page JB McCoy CB The effect of marital status on survival in late-stage cancer patients: an analysis based on surveillance, epidemiology, and end results (SEER) data, in the United States Int J Behav Med 1999 6 2 150 176 10.1207/s15327558ijbm0602_4 16250685

[B41] Beasley JM Newcomb PA Trentham-Dietz A Hampton JM Ceballos RM Titus-Ernstoff L Egan KM Holmes MD Social networks and survival after breast cancer diagnosis J Cancer Surviv 2010 4 4 372 380 10.1007/s11764-010-0139-5 20652435 PMC2978785

[B42] Kroenke CH Michael Y Tindle H Gage E Chlebowski R Garcia L Messina C Manson JE Caan BJ Social networks, social support and burden in relationships, and mortality after breast cancer diagnosis Breast Cancer Res Treat 2012 133 1 375 385 10.1007/s10549-012-1962-3 22331479 PMC4856003

[B43] Manjer J Carlsson S Elmstahl S Gullberg B Janzon L Lindstrom M Mattisson I Berglund G The Malmo diet and cancer study: representativity, cancer incidence and mortality in participants and non-participants Eur J Cancer Prev 2001 10 6 489 499 10.1097/00008469-200112000-00003 11916347

